# The emergence of scalar meanings

**DOI:** 10.3389/fpsyg.2015.00141

**Published:** 2015-02-19

**Authors:** Urtzi Etxeberria, Aritz Irurtzun

**Affiliations:** CNRS, IKER (UMR 5478)Bayonne, France

**Keywords:** prosody, semantics, focus, additivity, scalarity, Basque

## Abstract

This paper analyzes the emergence of scalar additive meanings. We show that in Basque the same particle *ere* can obtain both the “simple additive” reading (akin to English *too*) and the “scalar additive” reading (akin to English *even*) but we argue that we do not have to distinguish two types of *ere*. We provide evidence, by means of a production and a perception experiment, that the reading is disambiguated by means of prosody (the placement of nuclear stress), which is a correlate of focus. We argue that the scalarity effect is generated by the combination of two presuppositions (a focus-induced one and a lexical one) and the assertion of the sentence.

## 1. Introduction

Languages vary in the way they generate different additive readings. There are languages with particular lexical particles to express simple additive and scalar additive readings (*cf*. English *too* vs. *even*) but in Basque, the same particle, *ere*, is used to express simple additive as well as scalar additive values. Thus, in this language, a string like (1) with the same lexical items and word order can obtain either a simple additive reading and a scalar additive reading.

(1)    Jon ere etorri da.
        Jon ere come aux
        *Simple:* John came too.
        *Scalar:* Even Jon came.

In this paper we provide experimental evidence that the simple additive and the scalar additive interpretations are distinguished by means of prosody, which is a main correlate of information-structure. We report two (production and perception) experiments showing that prosody (in particular, association to nuclear stress and post-focal pitch compression) is what creates the scalar additive interpretation of the additive particle.

Besides, in order to account for how the scalar interpretation arises, we propose that the scalar interpretation of the particle *ere* is derived by combining the two presuppositions created by the sentence containing *ere*, i.e., the lexical-semantic contribution of *ere* and the focal presupposition, and the assertion of the sentence.

The paper is organized as follows: in Section 1.1 we briefly overview the semantic contribution of focus-sensitive operators like *even* in English. Section 1.2 presents the properties of the Basque particle *ere*, which can create both a simple additive and a scalar additive interpretation. In Section 2, we present the production and the perception experiments that we ran. In Section 3 we provide a novel analysis of the derivation of scalar interpretations from constructions with a lexically unambiguous simple additive particle, and finally, Section 4 concludes the paper.

### 1.1. The association with focus of *even*

Before we move on to see the properties of the Basque particle *ere* in Section 1.2, we will first concentrate on the English focus-sensitive operator *even*, on its semantic properties and on its contribution to the sentence it appears in. The semantics of *even* has been of great interest for linguists for some years now (*cf*. Jackendoff, 1972; Karttunen and Peters, 1979; Rooth, 1985, 1992; von Stechow, 1991; Wilkinson, 1996; Guerzoni, 2002; Giannakidou, 2007, *a.o*.). The literature agrees in treating it as a focus-sensitive operator. Take, e.g., the example in (2), where “Bill” is the associated element of the particle *even* and bears the focus feature (represented by the subscript F here and throughout):

(2)    John invited even [Bill]_*F*_.

In a sentence like (2), it is generally assumed that the focus-sensitive operator *even* is truth-conditionally vacuous (*cf*. Karttunen and Peters, 1979) and that the sentence has two main contributions: on the one hand it asserts “that John invited Bill” and on the other it provides two presuppositions: (i) the existential presupposition that “there are other x-s besides Bill such that John invited those other x-s,” *cf*. (3-a)^1^; and (ii) the scalar presupposition that “for all x-s under consideration besides Bill, the likelihood of John inviting those x-s is bigger than the likelihood of John inviting Bill,” *cf*. (3-b)^2^.

(3)    a.   Existential presupposition:
              ∃x [x ≠ Bill ∧ invited (j,x)].
        b.   Scalar presupposition:
              ∀x [x ≠ Bill → likelihood (John inviting x) > likelihood (John inviting Bill)]

Therefore, the contribution of the focus-sensitive particle *even* is to relate the asserted proposition to a set of alternative propositions (à la Rooth, 1985, 1992) which are obtained by substituting the element that bears the focus feature by its contextually relevant alternatives. These alternatives are ranked in a “likelihood” scale which gets its value by means of the context, e.g., scale of difficulty, scale of animosity, scale of friendship, etc. So, basically, the particle *even* contributes to the sentence by creating a scale of likelihood (e.g., scale of friendship) and by locating the asserted proposition, “that John invited Bill” in the case at hand, at the bottom of this scale.

(4)    John invited Mary >
        John invited Bryan >
        John invited Peter >
        John invited Bill

Interestingly, when *even* occurs in a negative context such as the one in (5) the scalar presupposition that we described in (3-b) is reversed, as shown in (5-b) (*cf*. Karttunen and Peters, 1979; Rooth, 1985, 1992; Wilkinson, 1996).

(5)    John did not invite even [Bill]_*F*_.
         a.   Existential presupposition:
               ∃x [x ≠ Bill ∧ ¬(invite j,x)].
         b.   Scalar presupposition:
               ∀x [x ≠ Bill → likelihood (John inviting Bill) > likelihood (John inviting x)]

Thus, in this case, the existential presupposition says that “there are other x-s besides Bill such that John didn't invite those other x-s” and the scalar presupposition says that “for all x-s under consideration besides Bill, the likelihood of John inviting Bill is bigger than the likelihood of John inviting those other x-s.” In other words, whereas in (2) Bill was the *least likely* person to be invited by John, in (5) the presuppositions are reversed and Bill is considered to be the *most likely* person to be invited by John.

In the next section, we will concentrate on the Basque particle *ere* and see what its properties and behavior are.

### 1.2. The basque particle *ere*

Now, let us turn our attention to the Basque additive particle *ere*. This particle is virtually unstudied [see some descriptions in Euskaltzaindia (1994), Hualde and Ortiz de Urbina (2003), and Ondarra (2007)].

From a syntactic point of view, *ere* can appear almost freely in any position of the clause, which, in neutral statements has the order S-IO-DO-V (6): following the subject (7), the indirect object (8), or the direct object (9):

(6)    Jonek Mireni liburu bat oparitu dio.
        Jon     Miren  book one offer    aux
        Jon offered Miren a book.
(7)    Jonek *ere* Mireni liburu bat oparitu dio.
        Jon    ere  Miren  book one offer    aux
        Jon too offered Miren a book.
(8)    Jonek Mireni *ere* liburu bat oparitu dio.
        Jon    Miren  ere  book one offer    aux
        Jon offered Miren too a book.
(9)    Jonek Mireni liburu bat  *ere* oparitu dio.
        Jon     Miren  book one ere  offer    aux
        Jon offered Miren also a book.

Some other contexts where *ere* immediately follows the lexical verb of a periphrastic construction are quite marked, and most speakers reject them as pertaining to a classical high register [*cf*. (10)]:

(10)   Jonek Mireni liburu bat oparitu *ere* dio.
        Jon     Miren  book one offer    ere  aux
        Jon also offered Miren a book.

However, it should be noted that *ere* cannot appear inside DPs (11), nor in sentence-initial position –given that it is an enclitic particle [*cf*. (12)]–, and that, in general, speakers also find it quite marked in sentence-final position (13):

(11)   ^*^Jonek Mireni liburu *ere* bat oparitu dio.
           Jon     Miren book ere one offer    aux
          Jon offered Miren a book too.
(12)   ^*^Ere Jonek Mireni liburu bat oparitu dio.
           ere  Jon     Miren book one offer    aux
         Also Jon offered Miren a book.
(13)    ^??^Jonek Mireni liburu bat oparitu dio   ere.
             Jon    Miren  book one offer    aux ere
            Jon offered Miren a book too.

Next, we will analyze the semantic contribution of *ere*.

### 1.3. Semantic contribution of *ere*

Regarding its semantic nature, *ere*'s core semantic contribution is that of a simple additive. Thus, a simple statement like (14-a) could coherently be followed by something like (14-b):

(14)    A.   Jonek Peru gonbidatu du.
                 Jon    Peru invite         aux
                 Jon invited Peru.
          B.   Aitor ere gonbidatu du.
                 Aitor ere invite        aux
                 He invited Aitor too.

Thus, we could picture *ere*'s contributed presupposition along the lines in (15), roughly, that “there are other x-s under consideration besides Aitor such that Jon invited those x-s”:

(15)   ∃x [x ≠ Aitor ∧ invite (Jon, x)]

Given this restriction, it is only natural that constructions requiring exhaustivity like (16)-(18) are ungrammatical with *ere*. In all of them, there is a clash between what the sentence asserts [the uniqueness restrictions of phrases like “*the* coach…” in (16), “the one that got the answer right…” in (17) or the cleft-like construction in (18)] and the additive presupposition introduced by *ere*^3^:

(16)   ^*^Taldearen entrenatzailea Regil *ere* da.
          team.of     coach            Regil ere  be
          The coach of the team is Regil too.
(17)   ^*^Erantzuna asmatu duen     bakarra Jon *ere* da.
          answer      figure   aux.C only       Jon ere be
          The only one that got the answer right is Jon too.
(18)   ^*^Jonek *ere* du     sagardoa erosi.
         Jon      ere  aux cider        buy
          Jon too is the one that bought cider.

However, the particle *ere* can also be employed to convey scalar additive values. Thus, a sentence with the same lexical items and word order of (14-b) can also have a scalar meaning, as represented in (19).

(19)   Aitor ere gonbidatu du.
         Aitor ere invite         aux
         (S)he invited even Aitor.

In this case, the presuppositions associated to *ere* are two, a simple additive one (20-a), and a scalar additive one (20-b):

(20)    a.   ∃x [x ≠ Aitor ∧ invite (Jon, x)].
                “There are other x-s under consideration besides Aitor such that Jon invited those x-s”.
          b.   ∀x [x ≠ Aitor → likelihood (Jon inviting x) > likelihood (Jon inviting Aitor)].
               “For all x-s under consideration besides Aitor, the likelihood that Jon invited those x-s is greater than the likelihood that Jon invited Aitor.”

In other words, it can also have the very same semantic import as English *even* (*cf*. Section 1.1) associating to the element preceding it. Likewise, under this reading the particle displays a similar behavior to that of *even* and, for instance, the scalar presuppositions brought up by *ere* are reversed under negation. Example (21) shows an instance of this reversal, whose corresponding presuppositions are presented in (22-a):

(21)   Jonek Aitor ere ez        du   gonbidatu.
         Jon    Aitor ere  neg    aux invite
         Jon did not invite even Aitor.
(22)   a.   ∃x [x ≠ Aitor ∧ ¬ invite (Jon, x)].
             “There are other x-s under consideration besides Aitor such that Jon did not invite x-s.”
         b.   ∀x [x ≠ Aitor → likelihood (Jon inviting Aitor) > likelihood (Jon inviting x)].
              “For all x-s under consideration besides Aitor, the likelihood that Jon invited Aitor is greater than the likelihood that Jon invited those x-s”

Hence, it would seem that phrases containing *ere* are completely ambiguous regarding the simple or scalar additive interpretations and that the listener would have to resort to discourse pragmatics in order to infer the correct interpretation of the sentence. What is more, it should be noted that *ere* is the only particle available in Basque to produce either simple additives or scalar additives [as opposed to other languages that have different items in the lexicon for different readings (*cf*. the references in Section 1.1)]. Notwithstanding, in this paper we will argue that this is not the case for Basque, i.e., that even if strings like (1) can correspond to the two readings, this is so just because out of any context written strings like (1) do not provide a representation of the intonation of the clause, and the information structure of the sentence is underspecified in the text. In fact, in Section 2 we will report the results of two experiments showing that prosody (nuclear stress placement) is a key factor in disambiguation^4^. From this observation, in Section 3 we will provide an analysis of the syntax-semantics-phonology interface proposing that the scalar additive reading derives directly from the simple additive reading and the information-packaging of the sentence.

## 2. An experimental analysis of the disambiguation of simple and scalar additivity

So far, we have argued that the particle *ere* can generate both simple additive and scalar additive interpretations, as shown in the examples in (23) and (24), whose only change is the interpretation of *ere*:

(23)   Amagoia ere eraman dute.
         Amagoia ere bring    aux
         They also took Amagoia.
(24)   Amagoia ere eraman dute.
         Amagoia ere bring    aux
         They even took Amagoia.

However, we will show that this potential ambiguity is just an illusion, and that prosody plays an important role in teasing apart the two readings. Thus, in order to test the variability in the interpretations of constructions with *ere* we designed two experiments. Experiment 1 is a production experiment designed to test the prosodic patterns associated to different readings (*cf*. Section 2.1.1) and Experiment 2 is a sentence-comprehension task where subjects had to judge the potential interpretations of utterances with *ere* with varying prosodic patterns (*cf*. Section 2.2). Then in Section 2.3 we briefly wrap up the main conclusions deriving from the two experiments; briefly, that different prosodic patterns (in particular, differences in the prosodic representation of information packaging) are associated to the different interpretations of the additive particle.

### 2.1. The production experiment

Experiment 1 was designed to assess the prosodic differences between sentences uttered with the simple reading in mind and sentences uttered with the scalar reading in mind.

#### 2.1.1. Experimental setting and participants

Experiment 1 is a laboratory phonology production experiment where 9 female participants (age *M* = 37.7, SD = 3.4), all native speakers of Central Basque (variety of Ordizia) were asked to utter, in as a natural way as possible, pairs of identical strings corresponding to simple additive and scalar additive interpretations^5^^,^^6^. In order to elicit the data, a presentation was shown in a laptop screen containing texts (written in the local dialect) that clearly favored one of the interpretations. There were three different strings, and two conditions per string which we term “Simple” and “Scalar”, all of them containing the same syllable in the accented positions in the element preceding the particle *ere* (/ru/) and the verb following it (/di/). All participants read the same set of sentences. Below we show the three strings (between brackets “< >”) and the six scenarios we employed to elicit them (here syllables /ru/, /re/, and /di/ are highlighted in boldface, but there was no such highlighting in the questionnaire presented to participants). Items (25-a), (26-a), and (27-a) are instances of the “Simple” condition and items (25-b), (26-b), and (27-b) instances of the “Scalar” condition:

(25)    a.   Mertxek azterketa gaindittu do. Eta *<I**ru**nek e**re** gain**di**ttu do>*.
               *English translation:* Mertxe passed the exam, and *<Irune* ere (=too) *passed the exam*.>
          b.   Irune klaseko txarrena da, askokatik gainea. Askotan pasatzen da klaseko danok azterketetan nota ona ateatzea eta beak suspenditzea. Halare, lehengon jarri ziguten azterketa hain erraza izan zan, *<I**ru**nek e**re** gain**di**ttu dola>*.
               *English translation:* Irune is, by far, the weakest in our class. Often times, we all get good grades and she gets an F. However, the exam that we got the other day was such an easy one that *<Irune ere* (=even) *passed the exam>*.
(26)    a.   Hegazkinaren istripuaren hotsa Hondarribian eta Lezon aditu da, eta *<I**ru**nen e**re** a**di**tu da.>*
               *English translation:* The sound of the plane-crash was heard in Hondarribia and Lezo, and *< it was heard in Irun* ere (=too) *>*.
           b.   AHTren obratarako Ezkion egiten ari diren leherketak normalean Ordizian, Tolosan eta asko jota Andoainen aditzen dira, baina lehengoan egin zuten leherketa hain handia izan zen *<I**ru**nen e**re** a**di**tu zela.>*
               *English translation:* The sounds of the explosions for the works of the high-speed train in Ezkio are normally heard in Ordizia, Tolosa and, at most, in Andoian. However, the other day they made such a big explosion that *<it was heard in Irun* ere (=even)*>*.
(27)    a.   Gaur goizean, soinketako 10 kilometroko frogan Jokin gelditu egin da. Eta *<Ma**ru**ri e**re** gel**di**tu da>*.
               *English translation:* In the 10 km running session of the gymnastics class this morning Jokin stopped. And *<Maruri* ere (=too) *stopped>*.
          b.   Normalen, soinketan, 10 kilometro korri egiten ditugunean, Iñaki Marurik bakarrik bukatzen du froga, beste guztiak gelditu egiten dira. Halere, gaur goizean jarri diguten frogan 10 kilometro mendian gora egin behar genituen, eta froga hain izan da gogorra *<Ma**ru**ri e**re** gel**di**tu dela>*.
               *English translation:* Normally, when we run 10 km in gymnastics, it is only Iñaki Maruri that finishes the race, all the rest stop at some point. However, in the race that they programmed for today we had to run 10 km up in the mountain, and the race was such a hard one that *<Maruri* ere (=even) *stopped>*.

Participants were asked to provide three repetitions of each string and condition so we got a total of 162 utterances (3 strings × 2 conditions × 3 repetitions × 9 speakers). In each of these utterances we took measurements in three syllables (/ru/, /re/, and /di/), so we analyzed 486 syllables in total (in a range of dimensions, as we explain below).

#### 2.1.2 Data and measurements

We measured syllable duration (in ms.), F0 mean and maxima (in Hertz), and intensity mean and maxima (in dB.) in the three syllables, as well as the F0 declination between F0 maxima in syllables /ru/ and /di/, which amounts to 5 measurements per syllable, and 16 measurements per utterance, to a total of 7776 measurements.

#### 2.1.3. Results^7^

We found significant differences between the two experimental conditions in both duration and F0 measurements^8^. Syllable /ru/ showed the same average duration in both conditions (*M* = 0.11, SD = 0.02), however, there were significant differences in the duration of syllable /re/ between the utterances on the Simple condition (*M* = 0.13, SD = 0.03) and those on the Scalar condition (*M* = 0.11, SD = 0.03), *t*_(80)_ = 5.24, *p* < 0.001, *r* = 0.51). Syllable /di/, like syllable /ru/ showed no significant duration difference.

**Figure 1:**
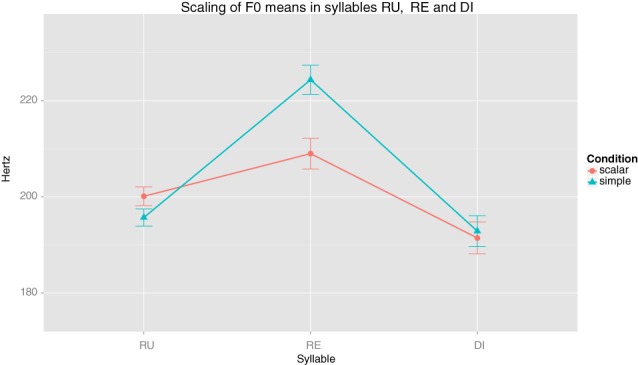
**Scaling of F0 means in syllables RU, RE and DI**.

In general, F0 values showed greater effects of the experimental manipulation. Observe, for instance, Figure 1, displaying F0 means in the three syllables that we measured.

As can be seen in the plot in Figure 1, on average, syllable /ru/ was pronounced with significantly higher F0 values in the Scalar condition (*M* = 200.12, SD = 17.65) than in the Simple condition (*M* = 195.70, SD = 16.16) (*t*_(80)_ = −2.85, *p* = 0.005, *r* = 0.3). On the other hand, syllable /re/ showed much higher F0 values in the Simple condition (*M* = 224.35, SD = 27.34) than in the Scalar condition (*M* = 208.99, SD = 28.65), *t*_(80)_ = 5.49, *p* < 0.001, *r* = 0.52). Last, at syllable /di/ no significant difference was observed between the Simple (*M* = 192.87, SD = 28.96) and the Scalar (*M* = 191.45, SD = 29.94) conditions; *t*_(80)_ = 1, *p* = 0.317, *r* = 0.112. However, it should be noted that even if maxima F0 values reached in syllable /ru/ under the Scalar condition (*M* = 210.29, SD = 20.29) are higher than those of the Simple condition (*M* = 209.07, SD = 17.38) a pairwise comparison of their means does not reach significance, which is probably due to the effect that the high values of syllable /re/ under the Simple condition make /ru/ keep high values overall. Last, declination between F0 maxima in syllables /ru/ and /di/ also showed significant effects, with the Simple condition showing a significantly smaller declination (*M* = 9.15, SD = 26.46) than the Scalar one (*M* = 14.11, SD = 23.09), *t*_(80)_ = −2.07, *p* = 0.041, *r* = 0.23. Converted into the logarithmic scale of semitones these measurements amount to *M* = 0.93 (SD 2.14), for the Simple condition, and *M* = 1.33 (SD 1.90) for the Scalar condition, a clear and perceptible difference (*t*_(80)_ = −2.17, *p* = 0.032, *r* = 0.24).

**Figure 2:**
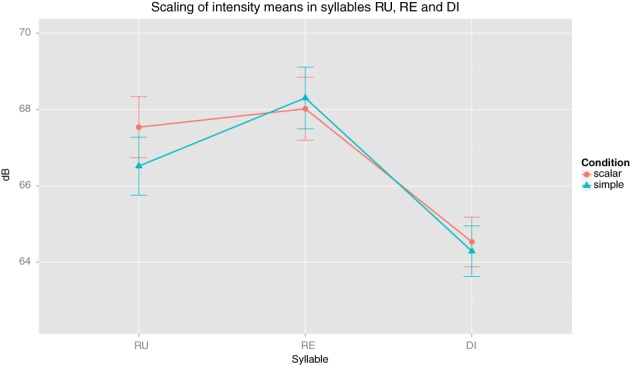
**Scaling of intensity means in syllables RU, RE and DI**.

Regarding intensity, both conditions were also distinguished (and note that this contrasts with previous studies on Central Basque intonation, which observed no correlation between nuclear stress and intensity values (*cf*. Irurtzun, 2013)). Observe as an illustration the plot in Figure 2, displaying intensity means in the three syllables.

On average, syllable /ru/ was pronounced with higher intensity in the Scalar condition (*M* = 67.54, SD = 7.21) than in the Simple condition (*M* = 66.52, SD = 6.84), *t*_(80)_ = −2.46, *p* = 0.017, *r* = 0.26. No difference was observed in syllables /re/ [Simple (*M* = 68.30, SD = 7.28), Scalar (*M* = 68.02, SD = 7.43)] and /di/ [Simple (*M* = 64.29, SD = 5.99), Scalar (*M* = 64.53, SD = 5.85)]. Comparison of maxima dB also shows significantly higher values at syllable /ru/ under the Scalar condition (*M* = 71.27, SD = 7.40) than under the Simple condition (*M* = 70.27, SD = 7.06) *t*_(80)_ = −2.39, *p* = 0.018, *r* = 0.25.

#### 2.1.4. Summary

The acoustic measurements discussed above show a clear difference between strings uttered in the Simple condition and strings uttered in the Scalar condition. And this is a remarkable fact, for the contexts of the utterance were unambiguous enough so that speakers would not convey any differences in their prosodic marking (that is, the exact interpretation of *ere* (simple vs. scalar) could be inferred from the context alone, but our observation is that even in this situation the tunes are different). In general, we saw that the stress associated to the element preceding the particle *ere* in the Scalar condition is stronger (in F0 and intensity) than in the Simple condition which, we would like to argue, is a signature of their focal nature (as narrow focus is associated to nuclear stress in Basque). Also, in the Scalar condition the region following this element displays reduced F0 values in comparison to the Simple condition, which would be linked to the well attested effect of post-focal pitch compression (*cf*. Elordieta, 1997, 2003; Elordieta and Irurtzun, 2009; Irurtzun, 2013; Hualde and Elordieta, 2014).

The conclusion of Experiment 1 is that speakers associate different prosodic patterns to different interpretations of the same string. In particular, strings associated to a scalar additive interpretation are characterized by having nuclear stress assigned to the element associated with *ere* (the element preceding it). Now the question that emerges is whether this intonational pattern is enough in and of itself to convey the intended meaning. That is, whether native speakers can identify the intended interpretation of each utterance. This is the goal of Experiment 2.

### 2.2. The perception experiment

Experiment 2 was designed to assess the interpretations associated to strings uttered with different prosodic patterns.

#### 2.2.1. Experimental setting and participants

**Figure 3:**
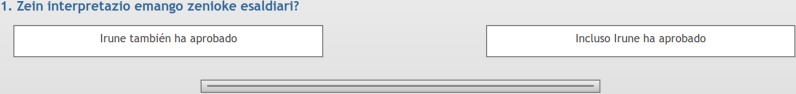
**A Visual Analog Scale (VAS) with Spanish unambiguous sentences at both ends**.

We designed a magnitude-estimation task with the help of a Visual Analog Scale (VAS) with unambiguous interpretations at both ends (see Figure 3). For the VAS, we took advantage of the fact that all Central Basque speakers are bilingual speakers of Spanish and Basque, and thus we designed a judgment task with unambiguous Spanish sentences at both ends (with *también* “also” and *incluso* “even”), as shown in Figure 3,^9^.

Thirty two Southern Basque speakers^10^ (21 female, age *M* = 31.6, SD = 8.9) were asked to listen to three strings which were uttered with two different interpretations in mind. Stimulus utterances were taken from the natural productions of a participant in Experiment 1. There were 3 test sentences in two conditions each (Simple and Scalar):

(28)   Irunek ere gainditu du.
         Irune   ere pass     Aux
         *Simple:* Irune too passed the exam [elicited in the context (25-a)].
         *Scalar:* Even Irune passed the exam [elicited in the context (25-b)].
(29)   Irunen     ere aditu da.
         Irun.Loc ere hear Aux
         *Simple:* It was heard also in Irun [elicited in the context (26-a)].
         *Scalar:* It was heard even in Irun [elicited in the context (26-b)].
(30)   Maruri ere gelditu da.
         Maruri ere stop    Aux
         *Simple:* Maruri stopped too [elicited in the context (27-a)].
         *Scalar:* Even Maruri stopped [uttered in the context (27-b)].

**Figure 4:**
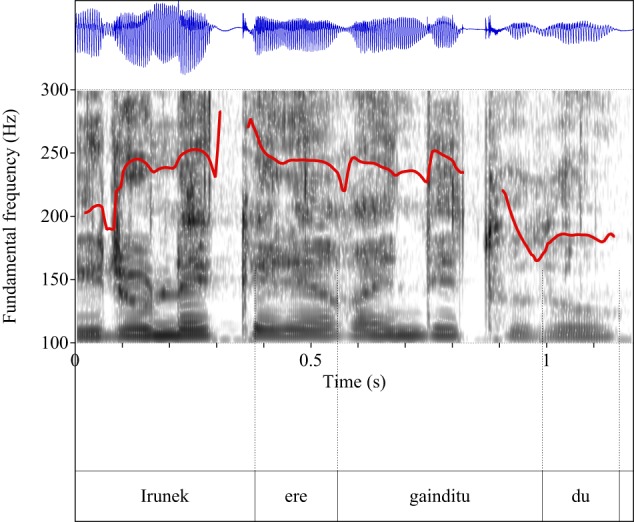
**Pitch track corresponding to test item (28), Condition: Simple**.

**Figure 5:**
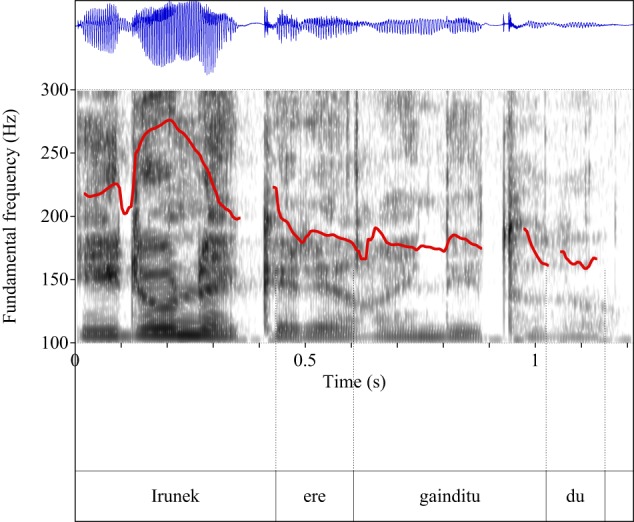
**Pitch track corresponding to test item (28), Condition: Scalar**.

**Figure 6:**
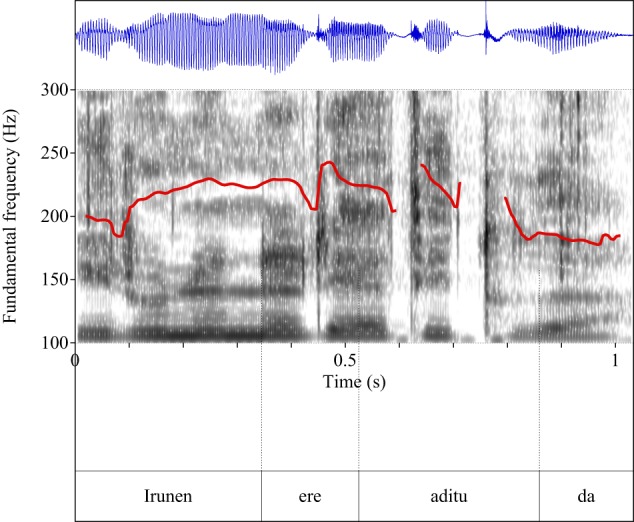
**Pitch track corresponding to test item (29), Condition: Simple**.

**Figure 7:**
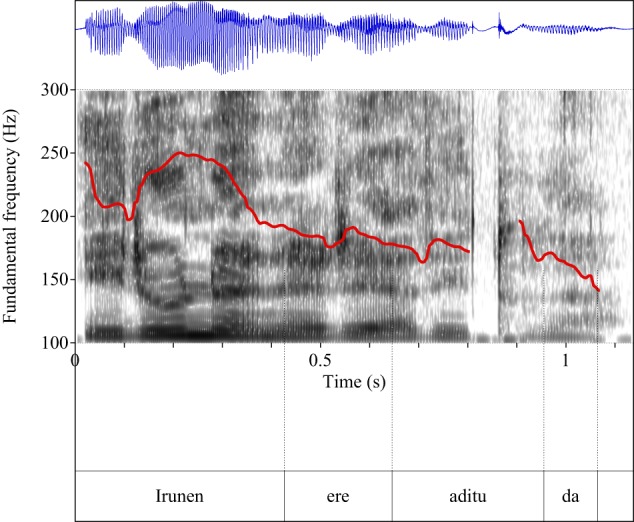
**Pitch track corresponding to test item (29), Condition: Scalar**.

**Figure 8:**
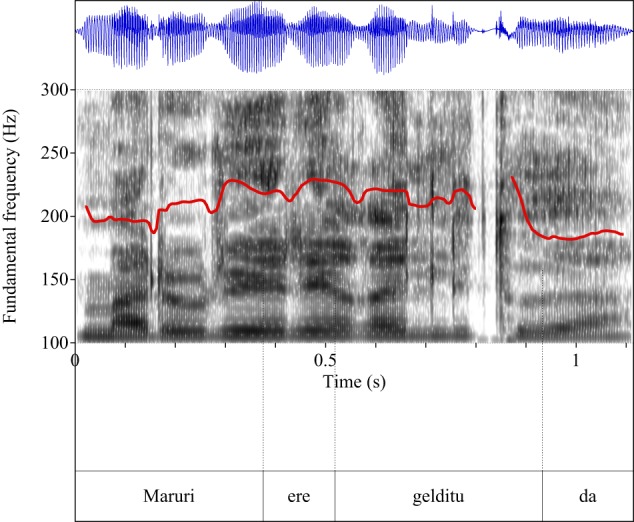
**Pitch track corresponding to test item (30), Condition: Simple**.

**Figure 9:**
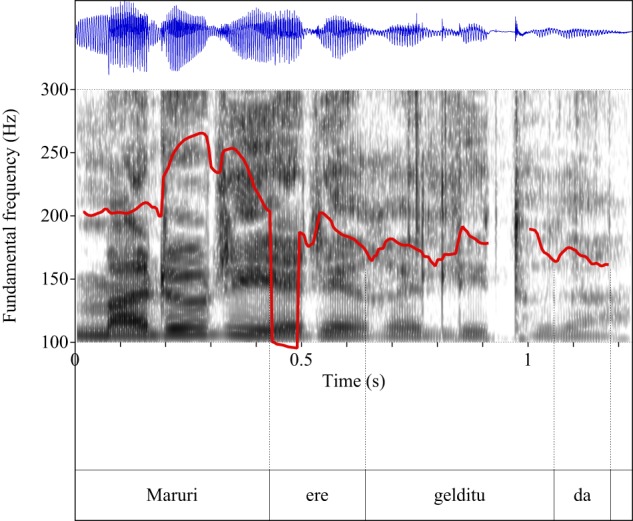
**Pitch track corresponding to test item (30), Condition: Scalar**.

Figures 4–9 show the pitch tracks corresponding to these stimuli (F0 in red over the spectrogram, waveform in blue).

**Figure 10:**
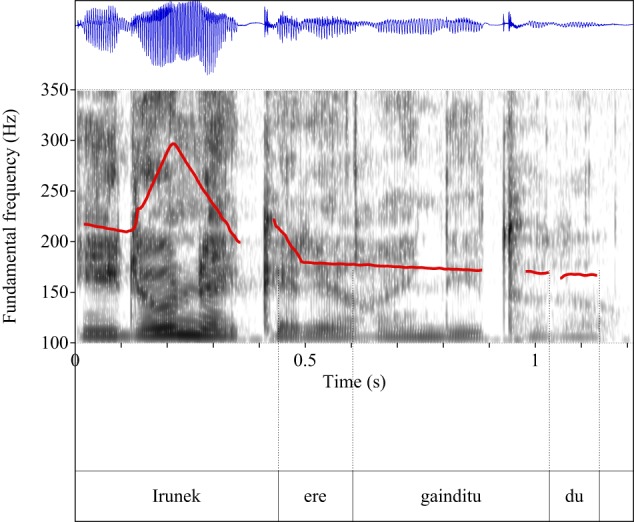
**Pitch track corresponding to test item (28), Condition: Synth1**.

**Figure 11:**
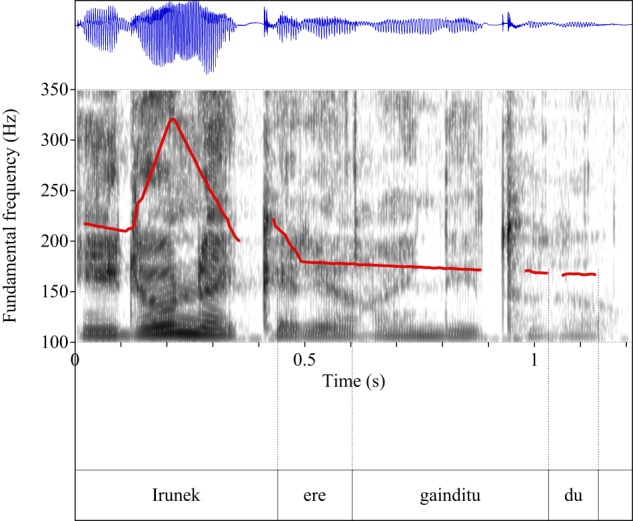
**Pitch track corresponding to test item (28), Condition: Synth2**.

Besides, for item (28), sentence “Irunek ere gainditu du” [(Even) Irune (too) passed the exam], we created an additional pair of test items: Condition Synth1, a manipulation of the item for “Scalar” by stylizing F0, raising the peak of the pitch accent in the subject by 25 Hz, and flattening the post-accentual region (Figure 10), and Condition Synth2, a manipulation of the item for “Scalar” by stylizing F0, raising the peak of the the pitch accent in the subject by 50 Hz and flattening the post-accentual region (Figure 11)^11^.

**Figure 12:**
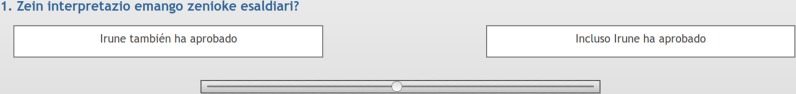
**A balanced VAS**.

Subjects listened to experimental items in isolation (i.e., with no context at all) and were instructed to judge the range of possible interpretations of each utterance in the VAS by cutting the judgment line in two: if they thought that the utterance was completely ambiguous and it could equally represent the two readings, subjects were instructed to place the delimiter in the middle of the line (as in Figure 12).

**Figure 13:**
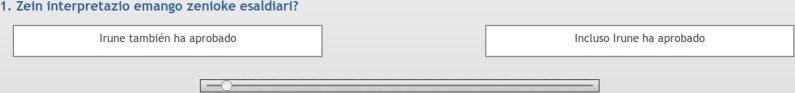
**A VAS aligned to the left**.

If they thought that it represented more the reading to the left, but still leaving some plausibility to the reading to the right they should place the delimiter on whichever place they felt on the left (see, for example Figure 13).

Alternatively, if they judged that the utterance was unambiguous in the other direction, they should place the delimiter more to the right. Subjects were explicitly instructed that they could place the delimiter at any point in the line. Besides the validity of the technique was controlled with completely unambiguous fillers that could only have one of the interpretations and hence should be placed at the extreme left or right boundary of the line. As we said, there were 8 test utterances in a questionnaire comprising a total of 40 utterances (the rest were fillers). Items were presented in a pseudo-randomized order, and in order to avoid any effect of a spatial-numerical association of response codes (*cf. i.a*. Dehaene et al., 1993) simple additive and scalar additive interpretations were presented both to the right and to the left of the VAS line in a random fashion (counterbalanced). Participants were instructed that they could listen to test items as many times as they wanted, but they could do just one mouse-click to cut the line, according to the interpretation they gave to the utterance they listened (the system was designed not to allow rethoughts or corrections). That is, once an utterance was evaluated, participants could not reevaluate it again.

#### 2.2.2. Data and measurements

**Figure 14:**
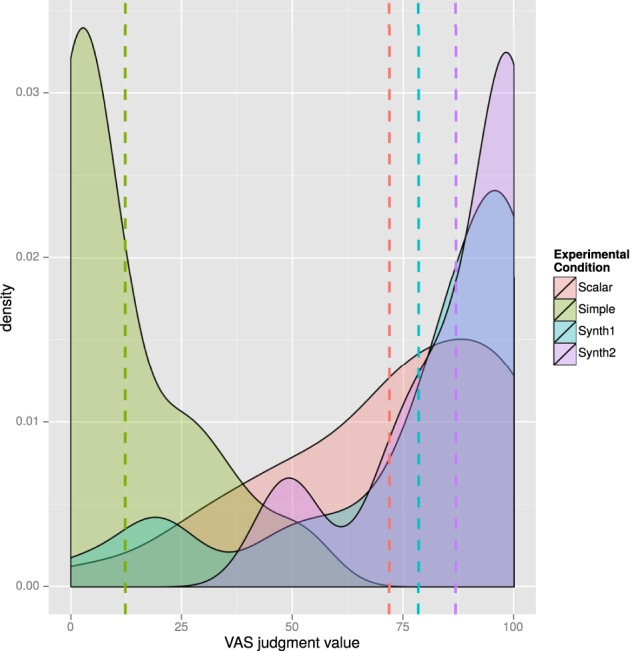
**Density plot of judgments for item (28) across four conditions**.

We measured participants' judgments on the VAS scale from 0 to 100 points (0, the value on the leftmost edge, 100 the value on the rightmost edge). Overall, judgments show a clearly skewed distribution. Figure 14 offers a view of the probability distributions (on the Y axis) of specific judgment values (on the X axis) according to the four different conditions of the test string “Irunek ere gainditu du” [(Even) Irune (too) passed the exam]. Dashed lines stand for mean values for each condition (Simple *M* = 12.31, SD = 15.58; Scalar *M* = 71.88, SD = 26.37; Synth1 *M* = 78.47, SD = 28.74 and Synth2 *M* = 86.88, SD = 17.30).

**Figure 15:**
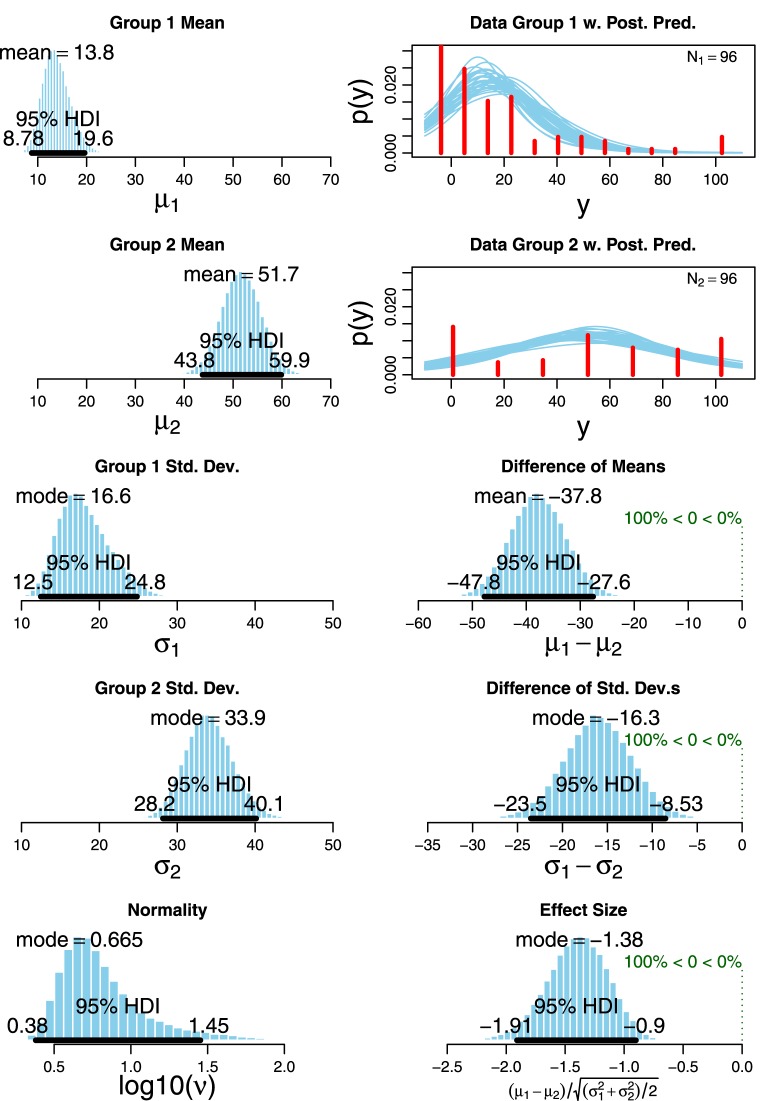
**Bayesian estimation: Simple (Group 1) vs. Scalar (Group 2)**.

As Figure 14 shows, responses to different conditions show a different behavior, with clearly skewed distributions, significantly so in the cases of conditions Simple, Synth1 and Synth2. In order to assess the robustness of the differences between conditions we departed from the classical null hypothesis significance testing (NHST) and performed a Bayesian estimation of differences between group means. In fact, many works have emphasized the limits of NHST methods like *t*-tests and their weakness *vis à vis* outlier data-points, which can affect greatly the analysis of the results of a sentence comprehension task like the one we designed (*cf. i.a*. Wagenmakers, 2007; Kruschke, 2011, 2013; Wetzels et al., 2011). In particular, we performed a pairwise comparison of the pooled judgments of Simple (*M* = 19.08, SD = 25.33) and Scalar (*M* = 50.78, SD = 35.70) items following Kruschke, 2013's BEST Markov chain Monte Carlo (MCMC) sampling method. Figure 15 provides an overview of the outcome of the Bayesian estimation of difference between groups for a MCMC sample of 100,000 parameter values.

As can be observed in the upper left panel, the mean of credible values for the mean of group 1 (i.e., the Simple condition) is 13.8, with the 95% highest density interval (HDI) ranging from 8.78 to 19.6, whereas the mean of credible values for Group 2 (the Scalar condition) is 51.7 with the 95% HDI between 43.8 and 59.9 (these are values of the MCMC posteriors). The difference between μ_1_ and μ_2_ is 37.8 on average, with 100% of the credible values well above zero. Thus, we can confidently conclude that the groups' means are indeed different (for comparison, the result of a paired *t*-test on these data is also clear: *t*_(95)_ = −6.59, *p* < 0.001, *r* = 0.56). Also, a credible difference is observed in the standard deviations of the two conditions (Simple Mo = 16.6 vs. Scalar Mo = 33.9), whereby the 100% of credible differences are greater than zero. Thus, not only is the mean of the Simple condition credibly smaller than the mean of the Scalar condition, but the standard deviation of the Simple condition is also credibly smaller than that of the Scalar condition, which means that, on average, items in the Simple condition are interpreted as simple additives and as less ambiguous than items in the Scalar condition, and that judgments for the Simple condition are more stable than those for the Scalar condition. The posterior also indicates that the effect size is large, since the histogram of the 100,000 credible effect sizes has a mode of −1.38 and a 95% HDI that excludes zero. As expected, analogous comparisons of the data for the other conditions in Figure 14 also showed sharp credible differences between the Simple condition and the rest (means in Figure 14 with dashed lines), since the judgments given by the participants shifts toward an unambiguously scalar interpretation with conditions with more marked accents (Simple < Scalar < Synth1 < Synth2).

### 2.3. Summary from the experimental results

In sum, our experiments show that constructions with *ere* can vary in their interpretations between the simple and the scalar additivity readings. However, this should not be interpreted as genuine ambiguity. In fact, Experiment 1 showed that the tunes associated to expressions with *ere* in contexts describing simple addition and scalar addition tend to differ; on average, the elements preceding *ere* in the scalar condition are associated with a focus intonation whereas in the simple condition they are not. This correlates with a clear shift in the interpretation of the sentence since, as Experiment 2 shows, stimuli extracted from the simple addition environments are clearly interpreted as simple additives, but items extracted from scalar addition environments shift their interpretation toward the scalar value, and the interpretation gets more scalar with stronger accents. Thus, we can conclude that there is a correspondence between the non-focal or focal nature of the element preceding the additive particle *ere* and the interpretation of the sentence as simple addition or scalar addition. The question is why? This is the issue that we tackle in the next section.

## 3. Proposal: the emergence of scalar meanings

In this section, we provide a novel analysis of how scalar interpretations of the Basque particle *ere* arise. In a nutshell, the analysis that we want to put forward has the following two ingredients: (i) the scalar value of *ere* is derived from the simple additive value of *ere*; in other words, we will only have a single lexical entry, i.e., the simple additive *ere*; (ii) the “least likelihood” reading or the “scalarity” derives directly from the combination of the two presuppositions of the sentence containing *ere* and the assertion of the sentence.

In order to account for the derivation of the scalar interpretation, i.e., the *even* reading, we will make use of the semantics of focus. Roughly, a main contribution of focus is the introduction of a presupposition to the effect that the property denoted by the sentence containing a focal element holds of some individual (*cf. i.a*. Geurts and van der Sandt, 2004), besides, focalization is generally taken to evoke “focus alternatives.” For instance, Rooth argues that a sentence which contains a focalized element has two denotations: (i) the “Ordinary Semantic Value” ([[Φ]]^*O*^) which is just the proposition denoted by the sentence, and (ii) the “Focus Semantic Value” ([[Φ]]^*F*^) that is a set of propositions obtained by the substitution of the focal phrase by “alternatives” that match it in syntactic and semantic types (*cf*. Rooth, 1985, 1992). So, for instance, the focused sentence in (31), which presupposes “that someone loves Paula,” would have the meaning represented in (32)^12^:

(31)     [Mary]_*F*_ loves Paula.
(32)              [[Φ]]^*O*^: {love(m, p)} = [[Mary loves Paula]]
                    [[Φ]]^*F*^: {love(x, p)∈ x ∈ E} = {[[Mary loves Paula]],
                    [[John loves Paula]], [[Peter loves Paula]], [[Sarah
                    loves Paula]], [[George loves Paula]]…}

That is, sentence (31) could be uttered in a situation where it is assumed that someone loves Paula, and we identify who that someone is by uttering (31), akin to saying that “it's Mary that loves Paula.” Now, with these ingredients in mind, how do we get the scalarity (least likelihood) reading for a sentence like (33) with the Basque particle *ere*?

(33)    [Jon]_*F*_ ere etorri da.
          Jon ere come    aux
          Even Jon came.

Our proposal is that the scalar interpretation of sentences like (33) derives directly from the combination of two types of presuppositions: on the one hand, we have the focal presupposition which forces us to reconstruct a context that presupposes “that someone came” (*cf*. Geurts and van der Sandt, 2004), and on the other hand, we also have the lexical-semantic contribution of *ere*, which when asserted generates the simple additive interpretation (34). The combination of these two presuppositions, one contextual (the focal one) and one lexical (the asserted simple additive), is what creates the complex presupposition “that someone came *and (s)he is not Jon*” (35). So this is a situation where we expect anyone but Jon to come. However, the expression of a sentence like (33) asserts “that Jon came,” clashing with our expectations (*someone came and (s)he is not Jon*), which is what brings about the counter-expectation reading that “Jon is the least expected/likely person to come,” i.e., the scalar meaning. It is the joint computation of the assertion and the presuppositions that generates this meaning.

(34)    ∃x [x ≠ Jon ∧ came (x)].
(35)    {came (x)| x ∈ E ∧ x ≠ Jon} = {[[Miren came and she is not Jon]], [[Peru came and he is not Jon]], [[Eneko came and he is not Jon]], [[Ane came and she is not Jon]]…}

In a nutshell, in these constructions an interpretation emerges where we expected anyone but Jon to come, and assert that Jon came, and this clash is what creates the counter-expectation reading (i.e., the scalar meaning).

## 4. Conclusions

In this paper, we have seen that in Basque the same particle *ere* can generate the “simple additive” reading as well as the “scalar additive” reading. In order to assess the potential ambiguity of constructions with *ere* we ran a production and a perception experiments and we concluded that prosody (in particular, nuclear stress and post-focal pitch compression) affects the interpretation of the additive particle. When the element preceding *ere* bears nuclear stress (i.e., when it is focal) the sentence gets a scalar interpretation.

Observing these facts, we have argued that the scalar value of constructions with *ere* is derived from the simple additive value of this particle, i.e., the simple additive value is the basic lexical meaning of this particle. The scalarity effect is generated by the combination of two presuppositions of the sentence containing *ere* [a lexical one (the lexical additive value of *ere*), and a focal one (the focal presupposition)] with the assertion of the sentence.

### Conflict of interest statement

The authors declare that the research was conducted in the absence of any commercial or financial relationships that could be construed as a potential conflict of interest.

## Footnotes

^1^We abstract from the discussion of whether the existential presupposition is needed in order to account for *even*'s semantic contribution or not (*cf*. Horn, 1972; Krifka, 1991; von Stechow, 1991). In fact, considering that we will be arguing in this paper that the primary meaning of the Basque particle *ere* is that of a “simple additive,” *cf*. Section 3, it is out of question whether this particle contributes the existential presupposition; it definitely does.

^2^Note that even if we employ the plural in the informal presentation of the scalar reading (“all x-s,” “those x-s,” *etc*.), the logical form of the presupposition in (3-b) does not imply any plurality; x could be a singleton.

^3^Constructions like (18) –which are specific to Navarro-Labourdin dialects–, are highly exhaustive constructions which in semantic terms are equivalent to clefts (*cf*. Duguine and Irurtzun, 2010), and hence, there is a sharp clash between the restriction of exhaustivity and the additive presupposition (as expressed in its English translation).

^4^In this paper, we use the term “disambiguation” in a non-technical sense. It does not imply that a structure that was ambiguous at derivational time *t*_*1*_ became unambiguous at derivational time *t*_*2*_; all it means is that prosody is the key factor unambiguously identifying the information structure of the multiple sentences (with different meanings) that could correspond to strings like (1), that is, that prosody is the key factor teasing apart which of the two readings is the actual one.

^5^We recorded 10 speakers in total, but the recordings of one of them had to be rejected before analysis given that she continuously stopped when reading.

^6^Our home institution does not require approval by any ethical committee for this sort of studies. This applies both to Experiment 1 and Experiment 2.

^7^All statistical tests were performed with R (R Development Core Team, 2008).

^8^See some pitch tracks in Figures 4–9.

^9^The experiment was conducted with the *LingMarket* website that we designed for data-gathering (URL: https://isqi.iker.univ-pau.fr/).

^10^All participants were L1 Basque speakers, or 2L1 Basque and Spanish speakers. All were fully competent in both languages. Also, they had to fill a short questionnaire before the experiment in order to assess that their linguistic capacities in Basque reached the European Framework C1 level.

^11^These test items were generated with Praat (Boersma and Weenink, 2014).

^12^Actually, we employ Rooth's formulation but any semantic theory of focus that assumes focal presuppositions will equally work for our proposal.
